# Bloodpool SPECT/CT offers enhanced sensitivity for the diagnosis of frozen shoulder

**DOI:** 10.1007/s12149-026-02246-5

**Published:** 2026-06-20

**Authors:** Maxence Gambiez, Emmanuel David, Claire Leplont, Charles-Louis Buee, Marc-Etienne Meyer, David Morland, Antoine Girard

**Affiliations:** 1 Department of Nuclear Medicine, Lille University Hospital, Rue Michel Polonowski, Lille, France; 2 Department of Nuclear Medicine, Amiens-Picardy University Hospital, Lille, France; 3 Orthopedic Surgery Department, Amiens-Picardy University Hospital, Amiens, France; 4 Department of Nuclear Medicine, Institut Jean-Godinot, Reims, France

**Keywords:** Bloodpool SPECT/CT, Shoulder pain, Adhesive capsulitis

## Abstract

**Objective:**

Adhesive capsulitis (frozen shoulder) is a common condition characterized by pain and progressive restriction of shoulder mobility, often requiring imaging for differential diagnosis. While bone scintigraphy is occasionally performed, the diagnostic potential of bloodpool SPECT/CT has never been evaluated.

**Methods:**

We retrospectively analyzed 18 affected shoulders in 17 patients who underwent standardized bone scintigraphy with both bloodpool and late-phase SPECT/CT. Uptake was visually assessed in four predefined capsular regions.

**Results:**

Bloodpool SPECT/CT revealed increased uptake in the rotator cuff interval in 100% of pathological shoulders, followed by the axillary recess (72%), anterior joint capsule (67%), and posterior joint capsule (44%). Late-phase diffuse uptake was present in 78% of cases. In three pathological shoulders, uptake in the rotator cuff interval was the only abnormality at the bloodpool phase.

**Conclusion:**

This study suggests that increased uptake in the rotator cuff interval on bloodpool SPECT/CT may be associated with adhesive capsulitis and could represent a sensitive imaging pattern. Further prospective studies are required to determine the diagnostic value of blood-pool SPECT/CT in patients with shoulder pain.

## Introduction

Frozen shoulder (adhesive capsulitis) is a common joint disorder affecting 2 to 5% of the population [1, 2]. It is characterized by pain and a gradual loss of both active and passive shoulder mobility, resulting from inflammation and adhesions within the glenohumeral joint capsule, significantly impacting daily life [1–6]. Early diagnosis is essential to initiate timely and appropriate multidisciplinary management [1, 7, 8]. Despite treatment, stiffness may persist in approximately 10% of patients [1].

The primary form, which has no apparent cause, contrasts with the secondary form, which is associated with general or local causes [2, 9, 10]. The latter raises challenges in distinguishing between differential or overlapping diagnoses with adhesive capsulitis. This is why imaging examinations are frequently used, including standard Xrays, ultrasound, arthrography, and magnetic resonance imaging (MRI) [2, 11–16], particularly in cases of therapeutic failure. These techniques help identify capsular and peri-capsular areas affected by inflammation and capsular retraction due to fibrosis. Such findings have also been observed in studies using [18F]Fluoro-2-deoxy-2- D-glucose ([18F]FDG) positron emission tomography/computed tomography (PET/CT) [17–19], although this modality is not usually recommended for diagnosing adhesive capsulitis. Even if many imaging modalities can help the clinician, clinical examination remains the reference standard for diagnosis [11].

Bone scintigraphy is one of the imaging techniques commonly used in clinical practice [20–22], although scientific evidence supporting its use in this indication is limited. However, no studies have yet evaluated the impact of recent procedures, such as the addition of bloodpool single photon emission computed tomography/computed tomography (SPECT/CT) to the standard bone scintigraphy protocol, for this indication. This early imaging phase allows the assessment of inflammatory processes in soft tissues, while the CT component enables anatomical localization. and characterization of pathological structures [23, 24]. This approach has demonstrated its relevance in various fields, such as the assessment of ankle and foot pain [25, 26], total knee prostheses [27], and osteomyelitis [28]. Drawing an analogy with these various techniques, our working hypothesis is that bloodpool SPECT/CT could more accurately identify inflammation of the shoulder synovial capsule.

The aim of this study is to evaluate the sensitivity of bone scintigraphy, including bloodpool SPECT/CT, for diagnosing adhesive capsulitis of the shoulder, and to identify the most sensitive patterns.

## Materials and methods

### Study population

In this monocentric study, we retrospectively included consecutive patients from March 2023 to December 2024 who were referred to our center for bone scintigraphy and met the following inclusion criteria: minimum age of 18 years old, diagnosed with adhesive capsulitis within 6 months before or after the bone scintigraphy, and no prosthetic material of the affected shoulder. All patients included in the study were informed and did not object to their participation in this study. This study was conducted in accordance with the principles set forth in the Declaration of Helsinki.

### Reference standard

The diagnosis of adhesive capsulitis was defined based on the following criteria: shoulder pain and stiffness for at least four weeks, severe pain impacting daily activities, nocturnal pain, limitation of both active and passive range of motion (anterior elevation 50°), and a normal radiographic appearance (for primary forms) [1].

### SPECT/CT protocol and analysis

The examinations were performed using two SPECT/CT systems: the Symbia Intevo 6 (Siemens) or the Discovery 870 DR (GE Healthcare). A standard activity of 8 MBq/kg of [99mTc] hydroxydiphosphonate was injected according to European Association of Nuclear Medicine (EANM) guidelines [29]. Each patient underwent imaging in two phases: (1) at the bloodpool phase, acquired 3 min after radiotracer injection, including SPECT/CT imaging of the shoulders, and (2) at the late-phase, acquired 3 h after injection, including whole-body static imaging and SPECT/CT of the shoulders. The bloodpool and late acquisitions were performed in 8 and 13 s per projection, respectively, for a total of 60 projections and a 180-degree rotation for each of the two detectors. The CT scans were performed with a slice thickness of 3 mm and without contrast agent. Additional acquisitions could have been performed at the physician’s discretion on the day of the examination. Images were analyzed simultaneously and consensually by two nuclear medicine physicians using Syngo.Via VB60 workstations (Siemens Healthineers, Erlangen, Germany).

### Images analysis

**Fig. 1:**
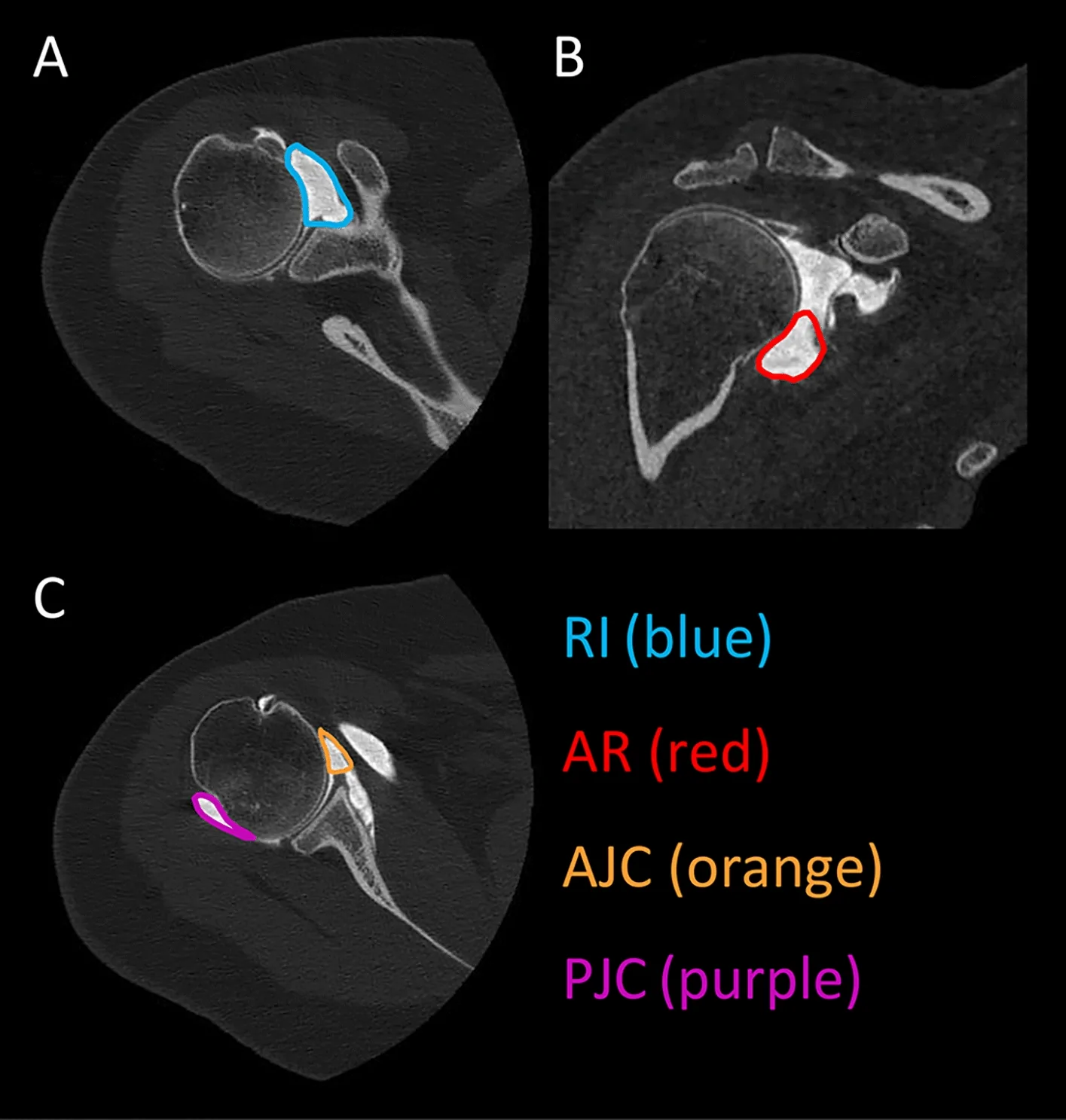
Capsular anatomy of the glenohumeral joint presented on an arthroscanner. RI, rotator cuff interval; AJC, anterior joint capsule; PJC, posterior joint capsule; AR, axillary recess

The analysis of each shoulder diagnosed with adhesive capsulitis was performed as follows. On the bloodpool SPECT/CT, four anatomical regions of the glenohumeral joint capsule were systematically evaluated for each patient: the rotator cuff interval (RI), the anterior joint capsule (AJC), the posterior joint capsule (PJC), and the axillary recess (AR) (Fig. 1). The retrospective image analysis was based on the findings from Kim et al., who studied metabolic patterns on [18F]FDG PET/CT in adhesive capsulitis [18]. A “diffuse uptake” was defined as the involvement of the four capsular regions (RI, AJC, AR, PJC) and extending beyond them to encompass the entire capsule. This clarification has now been added to the manuscript. On the late-phase SPECT/CT, image analysis focused on identifying a diffuse uptake pattern of the radiotracer within and/or around the humeral head [20]. Image interpretation was based solely on visual analysis by a consensus of two senior nuclear medicine physicians, assessing whether there was an increase in radiotracer uptake within each predefined location. The CT acquisitions coupled with the SPECT images allowed localization and morphological assessment of any underlying changes associated with the scintigraphic anomalies.

### Statistical analysis

Statistical tests and graphical representations were performed using MedCalc® version 12.5.0.0 (Medcalc Software bvba, Ostend, Belgium). The descriptive statistics of the population were reported according to their frequencies, their means accompanied by their standard deviations (SD), or their medians with interquartile interval [Q1,Q3]. The descriptive statistics of images were reported by proportions and 95% confidence interval (95%CI).

## Results

### Patients

A total of 18 shoulders were analyzed from 17 patients (9 men, median age of 54.9 years [51.5, 59.9]). The shoulder pains lasted for a median duration of 5.0 months [3.9, 10.9] when SPECT/CT were performed. Patients’ characteristics are detailed in Table 1. Only one out of 17 patients (6%) was referred by his general practitioner, while all the others (94%) were referred by rheumatologists or orthopedists. All imaging examinations were performed after the clinical diagnosis of adhesive capsulitis had been established, with a median delay of 2.4 months [1.4, 2.8]. No patients underwent multiple SPECT/CT analyses.

**Table 1:** Patients’ characteristics

	Number (%) or median [Q1, Q3]
Patients	n = 17
Sex (male)	9 (53%)
Sex (female)	8 (47%)
Age (years)	54.9 [51.5, 59.9]
Shoulders	n = 18
Laterality (right)	9 (50%)
Age of symptoms (months)	5.0 [3.9, 10.9]
Previous surgery	7 (41%)
Previous injury	9 (53%)
Delay between SPECT/CT and clinical diagnosis (months)	2.4 [1.4, 2.8]

### Scintigraphic patterns

Based on the standard late-phase SPECT/CT 14 out of 17 patients (78% [52%, 94%]) presented an increased uptake, as a diffuse articular or peri-articular uptake.

**Fig. 2:**
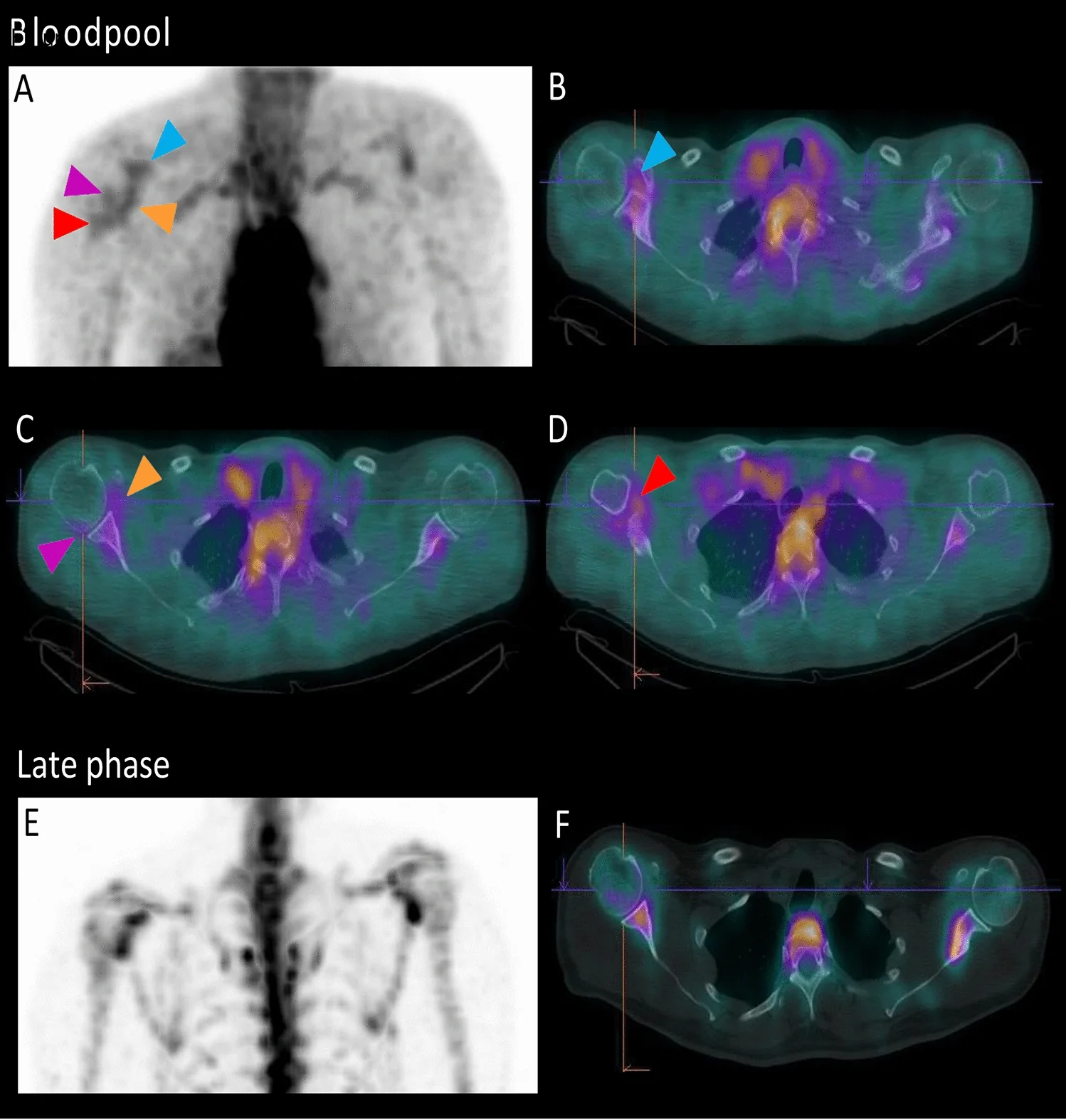
A fifty-three-year-old man presenting with clinical right frozen shoulder (patient 04R). Bloodpool phase SPECT/CT revealed an increased uptake in RI (blue arrow head) and AR (red arrow head) on the MIP (**A**) and fused images (**B** and **D**), while no significant uptake was observed in the AJC and the PJC (**C**). The late phase SPECT/CT, revealed symmetrical physiological uptake in the shoulders (**E** and **F**). RI, rotator cuff interval; AJC, anterior joint capsule; PJC, posterior joint capsule; AR, axillary recess; MIP, maximum intensity projection

**Fig. 3:**
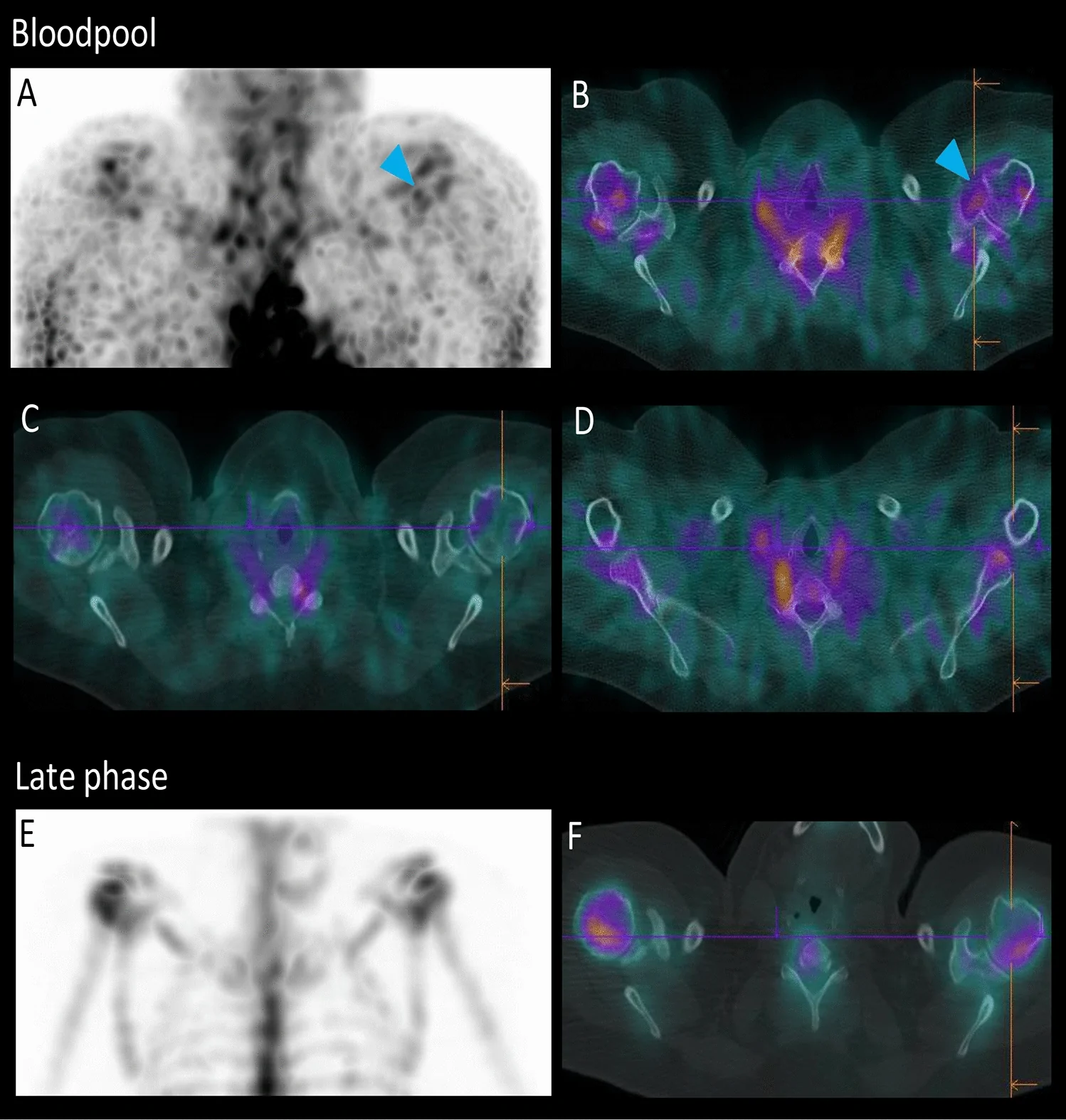
A forty-two-year-old man presenting with clinical left frozen shoulder (patient 01L). Bloodpool phase SPECT/CT showed an increased uptake in RI (blue arrow head) on the MIP (**A**) and fused images (**B**), while no significant uptake was observed on the AJC, PJC, and AR (**C** and **D**). The late phase SPECT/CT, revealed symmetrical physiological uptake in the shoulders (**E** and **F**). RI, rotator cuff interval; AJC, anterior joint capsule; PJC, posterior joint capsule; AR, axillary recess; MIP, maximum intensity projection

On the bloodpool SPECT/CT images, all the patients presented an increased uptake in the RI (100% [95%CI: 81%, 100%]). Additionally, 12 (67% [95%CI: 41%, 87%]) presented an increased uptake on the AJC, 13 (72% [95%CI: 47%, 90%]) on the AR, and 8(44% [95%CI: 22%, 69%]) on the PJC (Table 2, Fig. 2 and Fig. 3). No significant uptake was observed in pain-free shoulders.

**Table 2:** Scintigraphic patterns

	Bloodpool SPECT/CT					Late-phase SPECT/CT
	RI	AJC	AR	PJC	Diffuse uptake	Diffuse uptake
01L	+	-	-	-	-	-
02L	+	+	-	-	-	-
03R	+	-	+	-	-	+
04R	+	+	+	+	-	+
05R	+	-	+	-	-	+
06R	+	+	+	+	+	+
07L	+	+	+	-	-	+
08R	+	-	-	-	-	+
09L	+	+	+	+	+	+
10R	+	+	+	+	+	+
11L	+	+	+	-	-	-
12L	+	+	+	+	+	+
13L	+	+	+	-	-	-
14R	+	-	+	-	-	+
15R	+	-	-	-	-	+
16R	+	+	+	+	+	+
16L	+	+	+	+	+	+
17L	+	+	-	+	-	+
TOTAL	18	12	13	8	6	14
Sensitivity [95%CI]	100% [81%, 100%]	67% [41%, 87%]	72% [47%, 90%]	44% [22%, 69%]	33% [13%, 59%]	78% [52%, 94%]

RI, rotator cuff interval; AJC, anterior joint capsule; PJC, posterior joint capsule; AR, axillary recess

## Discussion

The objective of this pilot study was to evaluate the sensitivity of bone scintigraphy, including the bloodpool SPECT/CT, for the positive diagnosis of adhesive capsulitis of the shoulder, and to identify the most sensitive patterns. Eighteen clinically pathological shoulders were analyzed in 17 patients. During the bloodpool phase, increased uptake within the RI was observed in all clinically pathological shoulders, providing a sensitivity of 100% in our sample. As a comparison, the sensitivity of the increased uptake in the AR, AJC, and PJC during the bloodpool phase, were 72%, 67%, and 44%, respectively, and 78% regarding the diffuse uptake in the delayed phase. Overall, among all the 18 pathological shoulders, three showed increased uptake exclusively in the RI at the bloodpool phase (two of which also showed diffuse increased uptake in the delayed phase).

This study leads to two main findings. First, the absence of diffuse delayed uptake in a pathological shoulder does not definitively rule out the diagnosis of adhesive capsulitis. Second, the absence of increased uptake in the RI during the early phase was associated with a lower likelihood of adhesive capsulitis. The design of this study does not allow for the assessment of specificity of such patterns.

To the best of our knowledge, no previous study has been published investigating the usefulness of bloodpool SPECT/CT for the diagnosis of adhesive capsulitis.

Studied capsular areas are easily identifiable on CT scans, even without contrast agent. The RI is a triangular anatomical space located on the anterior aspect of the shoulder, bounded superiorly by the tendon of the supraspinatus muscle, inferiorly by the tendon of the subscapularis muscle, laterally by the long head of the biceps brachii muscle, and medially by the coracoid process. It is closed superiorly by the coracohumeral ligament and deeply by the superior glenohumeral ligament [30, 31]. Its inflammation in adhesive capsulitis is explained by synovitis, followed by fibrous collagen proliferation with synovial hypertrophy and deposits [30]. On MRI and MR arthrography, this fibrous tissue replaces the fatty signal on T1, thickens the capsule of the interval, as well as the superior glenohumeral and coracohumeral ligaments [14]. A T2 hypersignal is possible, as well as enhancement after gadolinium injection [32]. Thickening of the coracohumeral ligament is also found on ultrasound, where the tendon appears hypoechoic with Doppler hyperemia [33]. Involvement of the AR is also described, with it appearing underdeveloped and retracted [32].

The role of nuclear medicine in the assessment of adhesive capsulitis of the shoulder has been scarcely studied. Kim et al. studied the metabolic patterns in [18F]FDG PET/CT of the glenohumeral joint in adhesive capsulitis of the shoulder [18]. Twenty-two shoulders were studied in 21 patients affected by this condition. Sixteen shoulders showed enhanced uptake of the RI, the AJC, and the AR (pattern type I). Two exhibited increased uptake of the AJC and the AR (pattern type II), 2 had involvement only of the RI and AJC (pattern type III), and 2 had focal involvement of the RI or the AJC (pattern type IV). The uptake intensity was significantly higher in the RI, the AR, and the AJC compared to the non-pathological shoulder or the control group (40 shoulders in 20 patients without shoulder pain). Sridharan et al. showed that among 15 patients with focal uptake with [18F]FDG PET/CT of the RI or the inferior AR, 9 had a diagnosis of adhesive capsulitis [19]. The sensitivity and specificity of [18F]FDG PET/CT for detecting adhesive capsulitis were 56% and 87%, respectively, with a likelihood ratio of 6.3 (95% CI: 2.8 – 14.6). Finally, Won KS et al. demonstrated a correlation between [18F]FDG PET/CT metabolic patterns and clinical involvement in 35 patients with adhesive capsulitis [17]. The uptake intensity value in the RI, AR, and AJC was negatively correlated respectively with external and internal rotation, abduction and elevation, and all movements.

The presence of increased uptake in the tissue phase of SPECT/CT thus seems to reflect, in our study, this inflammatory environment, mainly found in the RI, then the AR.

Some limitations have to be considered in our study. First, it was a monocentric study with a limited sample size that hampered the statistical power. Second, due to the imperfect spatial resolution of SPECT, the analysis of subcentimetric anatomical structures may be imprecise as a consequence of the partial volume effect. Third, due to the retrospective nature of the study, the diagnostic assessment was based on a review of consultation reports written by orthopedic specialists, rather than standardized reassessment, which did not consistently provide detailed descriptions of the extent or severity of the condition. Consequently, it was not possible to establish a precise correlation between imaging findings and clinical severity, and heterogeneity in the reference diagnosis of adhesive capsulitis may have been introduced. Prospective studies using standardized clinical criteria are needed to confirm these findings. Finally, the specificity and positive predictive value of RI uptake could not be assessed in this study, as no control group—including shoulders without adhesive capsulitis or with alternative diagnoses—was included. Therefore, although all confirmed cases showed increased uptake, we cannot conclude that RI uptake is specific to adhesive capsulitis or that its presence reliably confirms the diagnosis.

While bone scintigraphy has not traditionally been considered in the evaluation of adhesive capsulitis, our study indicates that bloodpool SPECT/CT may offer valuable diagnostic support. Additional studies are required to complete this pilot study, first to validate our findings and clarify the specificity of the described patterns in larger cohorts including other pathologies of the shoulder, as well as to determine its role in therapeutic decision-making and its correlation with patient outcomes. Its position among other imaging techniques should also still needs to be clarified. The clinical impact of the bloodpool SPECT/CT, that has been demonstrated in several clinical settings [25–28, 34] could be enhanced through the use of more advanced systems such as full-ring 360° cadmium zinc telluride (CZT) technology [35].

## Conclusion

Bloodpool SPECT/CT may represent a promising imaging modality for the evaluation of shoulder adhesive capsulitis. In this preliminary study, increased uptake in the RI on bloodpool SPECT/CT was observed in all shoulders diagnosed with adhesive capsulitis. These findings should be interpreted cautiously and require confirmation in larger prospective controlled studies.
